# Comparative Response of the West African Dwarf Goats to Experimental Infections with Red Sokoto and West African Dwarf Goat Isolates of* Haemonchus contortus*

**DOI:** 10.1155/2015/728210

**Published:** 2015-11-30

**Authors:** Lucas Atehmengo Ngongeh, Amaechi Onyeabor

**Affiliations:** Department of Veterinary Microbiology and Parasitology, Michael Okpara University of Agriculture, Umudike 7267, Abia, Nigeria

## Abstract

Response of the West African Dwarf (WAD) goats to two different isolates of* Haemonchus contortus*, the Red Sokoto (RS) goat isolate (RSHc) and the WAD goat isolate (WADHc) (isolated from WAD goats), was studied by experimental infections of 4–6-month-old male WAD goat kids. Group 1 and Group 2 goats were each infected with 4500 infective larvae (L3) of RSHc and WADHc, respectively. Group 3 animals served as uninfected control. Prepatent period (PPP), faecal egg counts (FEC), worm burden (WB), body weight (BW), packed cell volume (PCV), and body condition score (BCS) were determined. WAD goats infected with RSHc isolate and the ones infected with WADHc isolate had mean PPP of 19.63 ± 0.26 and 19.50 ± 0.19, respectively. Goats infected with WADHc isolate had significantly higher FEC (*P* = 0.004) and WB (*P* = 0.001). BW were significantly higher (*P* = 0.004) both in the controls and in Group 2 goats infected with WADHc isolate than in Group 1 goats infected with the RSHc isolate. BCS of animals in both infected groups dropped significantly (*P* = 0.001). There was a significant drop in PCV (*P* = 0.004) of both infected groups in comparison. Both isolates of* H. contortus* were pathogenic to the host.

## 1. Introduction

The importance of animal production cannot be overemphasized as the industry plays vital roles as a great source of protein and economic earnings amongst others. As an example, the WAD goats play a useful role in the rural economy of West African states, particularly the small-scale and marginal livestock owners [1]. Ruminants constitute a large proportion of the livestock industry; however, their productivity is limited by various factors such as helminthosis, particularly haemonchosis, caused by members of the genus* Haemonchus*.* H. contortus* is the main aetiology of PGE which is an important syndrome that seriously affects small ruminant production. The most popular method of control of helminthosis is the use of anthelmintics. Unfortunately, the widely increasing rate of development of anthelmintic resistance to most of the commercially available anthelmintics has become a big source of worry to the future of small ruminant production in particular and livestock in general [2]. Part of the main reasons why the WAD goats thrive and have remained popular in the region in spite of their small sizes may be the fact that they are trypanotolerant and about 85% of the animals are naturally endowed with the potential to resist haemonchosis and for such reason they have been labelled haemonchotolerant [3, 4]. The ability of the WAD goats to tolerate trypanosomosis and haemonchosis in the region certainly has been drawing attention to the reason why the animals survive despite the little or no chemotherapeutic control of the infections.

A lot of research efforts are being made to salvage the industry from the serious threat, such as selection and development of resistant breeds against the infections. Such efforts include the series of studies conducted over ten years in Nigeria by the Sir Halley Stewart Trust (SHST) Project, a collaborative research team involving University of Nigeria, Nsukka, in Africa, University of Nottingham in the UK, and the Wakwa Regional Research Institute, ILRAD, Cameroon, in Africa. The earlier studies have reported the trypanotolerant and haemonchotolerant nature of WAD goat [1, 3, 4]. This potential was deemed amazing and the researchers have long attributed it to a sort of genetic endowment, although the genes that may be responsible are yet to be identified [1, 3, 4]. However, it was also hoped that other factors such as the influence of parasite isolate or strain could also partially account for such unique responses of the WAD goats and they are also to be investigated in order to fully describe the uniqueness of that breed of goat.

There is need, however, to increase the strides and one of the most important ways of achieving this is to update the knowledge about helminth parasites and how they act in their hosts or how their hosts respond to their presence. It is only in this way that parasite control efforts can be made to be a step ahead of the challenge, as this can enable us to adapt better ways of drug administration, either singly or as part of an integrated parasite control system (for efficient control methods). To do this, however, the helminth parasites and helminth parasite-host interactions should be explored not only at the species level, but various strains and isolates of the important parasites should be studied in various environments. There are pointers that lots of different isolates of parasites exist with genetic variation [5]. For example, significant genetic and phenotypic variation exists between isolates of* H. contortus* in Australia [6].

Enormous within-population variations, low genetic differentiation, and high gene flow among different populations of* H. contortus* have also been revealed in China [5]. In addition, three isolates of* H. contortus*, namely, autochthonous (Aran99) and two allochthonous (MRI and MSD), have been shown to cause different levels of pathology in a primary infection in Manchego lambs [2]. The potential of different isolates of helminths behaving differently has also been exhibited by the fact that different isolates of* H. contortus* respond differently to different anthelmintics with some being susceptible while others are not due to the development of anthelmintic resistance [7]. For example, some field isolates of* H. contortus* have been shown to have higher resistance to ivermectin than others [8]. It has been reported that Malaysian sheep and goat populations share the same isolate of* H. contortus* unlike in Yemen where different isolates may be found and this was suggested to be taken into account in the design of an effective control strategy [9]. It has been stressed that knowledge of genetic variation within and among* Haemonchus* populations can be a springboard for understanding the transmission patterns and spread of drug resistance alleles and might assist in the control of haemonchosis [10]. Consequently, the objective of this study was to compare the response of the WAD goat to single primary infections of either RS or WAD goat isolates of* H. contortus*. It was hoped that the results would help inform decisions as whether the two goat breeds could be mix-grazed on same pastures or whether to encourage the dominance of a particular* H. contortus* strain on pasture based on the response of the host to the isolates. It was also hoped that the results would constitute part of the baseline data for the tracking of anthelmintic resistance in the country.

It would be a welcome issue if the WAD goat responds similarly to both isolates of* H. contortus* so that both breeds of goats can be reared together without serious threats of pathology posed by one strain of the parasite to other hosts. This is partly because interbreeding between the WAD and RS goats and other larger goat breeds is being practiced by some WAD goat keepers to obtain larger sized offspring as a means for the very small size of the WAD goat and partly due to the influx of the RS goat from the northern part to the eastern part of Nigeria for sales. However, limitations such as the inability of one breed to cope with the worms from the other breed could forestall such effort. In a situation where one strain of* H. contortus* is to be more pathogenic, the milder one could be encouraged to predominate in the population since this would cause less pathology in the hosts or better still the milder one could be used as a vaccine for the prevention of the infections.

## 2. Materials and Methodology

### 2.1. Experimental Animals

The study was conducted at Michael Okpara University of Agriculture, Umudike (MOUAU), in Abia. Four- to six-month-old male WAD goat kids were used for the study. Apparently healthy goats were purchased from local markets around Umuahia. Following purchase, the goats were routinely treated against ectoparasites and endoparasites as described by Fakae et al. [11]. Briefly, on arriving at the animal house in MOUAU, the goats were tethered outside the quarantine and treated with an acaricide, anticoccidial drug, and anthelmintic to rid them of ectoparasites, coccidia, and GI nematodes. They were also vaccinated against peste des petits ruminants (PPR) using tissue culture rinderpest vaccine (TCRV, NVRI, Vom, Nigeria) before introducing them to the quarantine where they were monitored for some days before transferring them to the experimental pens where they were acclimatized for four weeks; within such time all the routine treatments were also completed. The goats were fed daily with fresh cut and carry grass and a supplement concentrate mixture comprising palm kernel cake and grower's mash as described by Fakae et al. [11]. They were given water* ad libitum*. The pens were all screened against flies.

The study complied to all relevant laws and codes of practice governing the experimental studies with life animals as stipulated by Ward and Elsea [12], and the experimental protocol was approved by Animal Ethics Committee of Michael Okpara University of Agriculture, Umudike.

### 2.2. Experimental Design

Following the acclimatization, the animals were randomly assigned to three groups, namely, Group 1 (*n* = 10), Group 2 (*n* = 10), and Group 3 (*n* = 10) (Table 1). Group 1 and Group 2 animals were each given single pulse infections of 4500 infective larvae (L3) of RS isolate (RSHc) and WAD isolate (WADHc) of* H. contortus*, respectively, per animal on day zero (D0) of the study and Group 3 served as the uninfected control. Faecal samples were collected daily (for FEC) from day 15 of infection until patency was achieved. Following patency, FEC were conducted twice weekly. Body weight (BW), body condition scoring (BCS), and packed cell volume (PCV) were carried out weekly till D56 when the experiment ended. The goats were humanely sacrificed on day 56 for the recovery of abomasal worms for worm counts.

### 2.3. *Haemonchus contortus* Infections

The RS and WAD goat isolates of* H. contortus* were used for the infections. In order to obtain the RSHc isolate, abomasae were collected from slaughtered RS goats recently brought from northern Nigeria. On arrival at the laboratory the adult female* H. contortus* were harvested from the abomasae and crushed to release the worm eggs. The egg suspension was then mixed with sterile faeces and the cultures were set in Petri dishes at room temperature. The larvae were harvested on the seventh day of culturing. Two helminth-free six-month-old male WAD goat kids were then infected with the larvae to serve as the donor goats for the RSHc isolate. To obtain the WADHc isolate, abomasae were collected from slaughtered WAD goats from eastern Nigeria and the adult female* H. contortus* were harvested and cultures were set as described initially. Two other six-month-old male WAD goat kids were also obtained and infected to serve as donors for the WADHc isolate. The donor goats for RSHc and WADHc isolates were kept in separate pens to avoid contamination of the isolates with each other through cross infection. The RS and WAD goat isolates of* H. contortus* infective larvae (L3) for the infections were harvested from faecal cultures prepared from the faeces of donor goats as described by Musongong et al. [13]. Larvae were preserved in the refrigerator (4°C) until being used for infection within two weeks. The estimated dose (4500 L3) was administered orally to each of the goats with the aid of a stomach tube.

### 2.4. Faecal Egg Counts

Faecal egg counts were conducted on fresh faeces collected from each goat per rectum or from clean paved floors where a goat was tethered away from other goats to defaecate. The counts were conducted by the flotation technique using saturated salt (sodium chloride) solution by following the modified McMaster technique [14, 15].

### 2.5. Worm Counts

On day 56 after infection when the experiment was terminated, the goats were humanely sacrificed and the abomasal worms collected for identification and counting. The abomasal worm counts were performed as described by Hansen and Perry [15] (1994).

### 2.6. Packed Cell Volume

The packed cell volume (PCV) was calculated using blood obtained from the jugular vein on day zero (D0) and subsequently every seven days till the end of experiment.

### 2.7. Body Weights

The weights of the goats were determined by weighing each goat separately on D0 using a weighing balance (Camry Emperors, China) and subsequently weekly as described by Fakae et al. [11] (1999).

### 2.8. Body Condition Score

The body condition score (BCS) of each goat was obtained by physically assessing the level of muscling and fat deposition over and around vertebrae in the loin region on day 0 as described by Russel [16] (1991). Subsequent BCS readings were taken weekly.

### 2.9. Statistical Analysis

Statistical analysis was conducted using SPSS version 15 for Windows. Parameters recorded on more than a single day were analysed by repeated measures analysis of variance (ANOVA) in General Linear Model (GLIM) and those recorded on a single day were analysed by one-way analysis of variance (ANOVA). Faecal egg counts were log 10 (FEC + 10) transformed prior to analysis to normalize the distribution. Paired-sample Student's *t*-test was also used where applicable. Summary data are presented as mean ± standard error of the mean (SEM) and probabilities (*P*) of 0.05 or less were considered significant.

## 3. Results

### 3.1. Faecal Egg Counts

**Figure 1:**
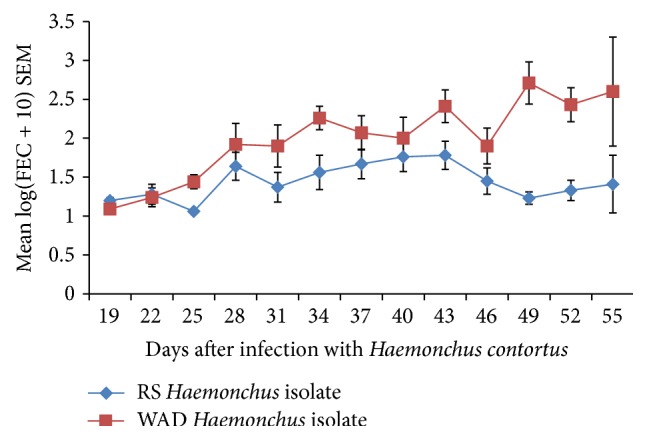
Mean strongyle egg counts of Nigerian West African Dwarf (WAD) goats given a single pulse infection with either a Red Sokoto (RS) or a WAD goat isolate of* H. contortus*.

The mean PPP was 19.63 ± 0.26 and 19.50 ± 0.19 for RSHc and WADHc infected goats, respectively (range: 19 to 20 days). There was no statistically significant difference between the prepatent periods (*P* < 0.298). Faecal egg counts generally rose in a similar pattern in both infected groups, being significantly higher in the goats infected with the WADHc isolate (*P* = 0.004). However, from D34, while FEC continued to rise in the WADHc isolate infected goats, FEC dropped in the RSHc isolate infected group till day 56 when the experiment ended (Figure 1).

### 3.2. Worm Burden

**Figure 2:**
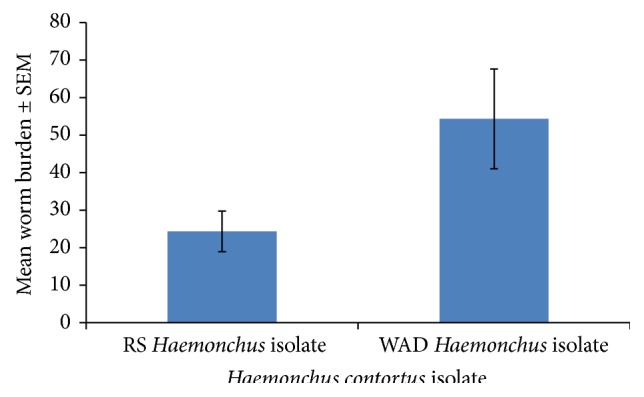
Mean worm burden of Nigerian West African Dwarf (WAD) goats given a single pulse infection with either a Red Sokoto (RS) or a WAD goat isolate of* H. contortus*.

The WB had the same trend as the FEC being generally low but significantly higher (*P* = 0.001) in the WADHc isolate infected group than in the RSHc isolate infected group (Figure 2).

### 3.3. Body Weights

**Figure 3:**
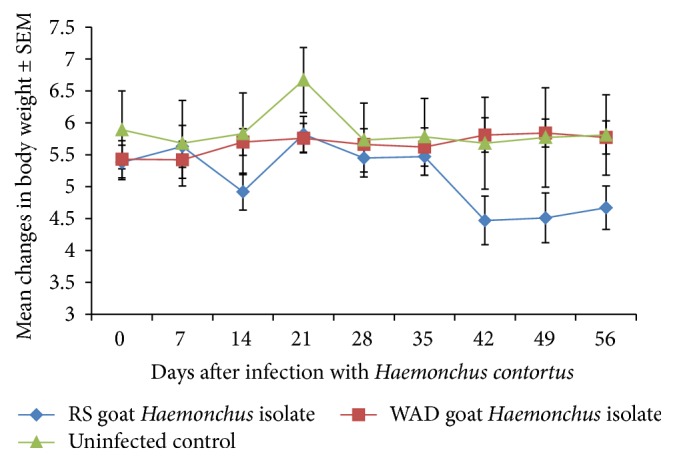
Mean body weights of Nigerian West African Dwarf (WAD) goats given a single pulse infection with either a Red Sokoto (RS) or a WAD goat isolate of* H. contortus*.

Goats infected with the* Haemonchus* isolates lost weight from D21 to D35 and the weight loss continued thereafter from D35 only in the WAD goats infected with WAD isolate of* H. contortus* (Figure 3). There was a significant weight loss of the RSHc infected goats from day 35 (*P* = 0.004) compared to the uninfected and WADHc infected goats.

### 3.4. Body Condition Scores

**Figure 4:**
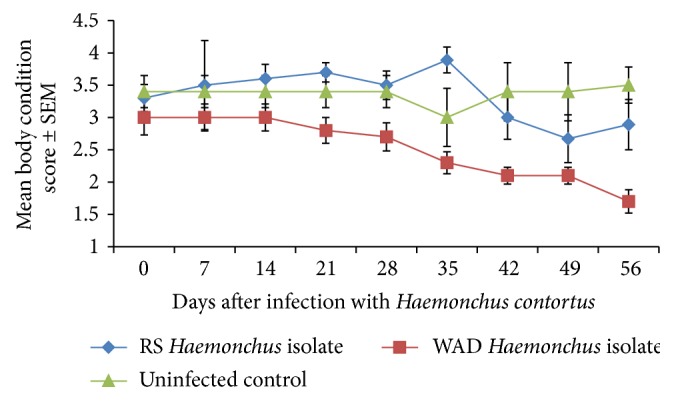
Mean body condition score of Nigerian West African Dwarf (WAD) goats given a single pulse infection with either a Red Sokoto (RS) or a WAD goat isolate of* H. contortus*.

The mean BCS of both infected and control goats generally improved from the start of the experiment up to day 14. However, infected goats have begun to lose body condition by day 21 compared with the uninfected controls, although there was a brief fall in mean BCS of the controls from day 21 to day 28 before rising from day 35 to day 56 (Figure 4). The lowering of mean BCS continued to the end of the study with the mean BCS of infected animals being significantly lower than that of the controls (*P* < 0.001). The drop in mean BCS was severe in both infected groups, although it started earlier (D7) in the goats infected with the WADHc isolate compared to those infected with RSHc isolate.

### 3.5. Packed Cell Volume

**Figure 5:**
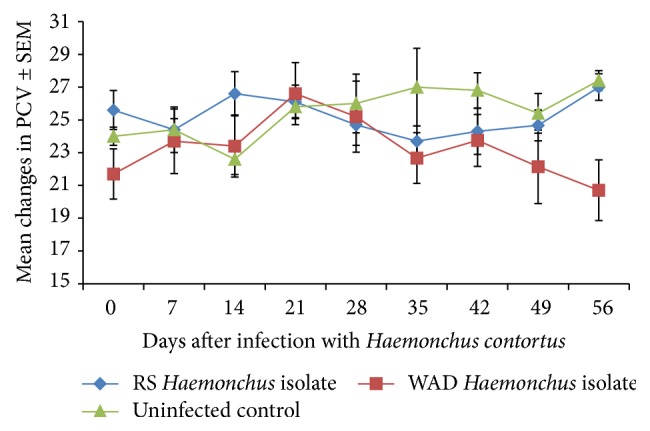
Mean PCV of Nigerian West African Dwarf (WAD) goats given a single pulse infection with either a Red Sokoto (RS) or a WAD goat isolate of* H. contortus*.

The PCV generally fluctuated. The fluctuations in PCV were more obvious in the RSHc infected goats with major declines in PCV on day 14 and day 42. This was in contrast to mild fluctuations in the WADHc infected goats although their PCV ended with a drop on day 56 (Figure 5). The drop in PCV was significantly lower (*P* = 0.004) in the infected goats in contrast to the uninfected control goats.

## 4. Discussion

The WAD goats were susceptible to both isolates of* H. contortus*. The two isolates of* H. contortus* used for the study established and produced patent infections, which were pathogenic to the WAD goats. The patency was evidenced by the worm eggs excreted in the host faeces (FEC), while the pathogenic effects were demonstrated by the low PCV, BW, and BCS of infected animals when compared with the uninfected controls. The relatively short PPP recorded in the infections with both isolates may partly be due to the young age of the animals; however, the PPP were still within the normal range for* H. contortus*, 17/19 to 25 days [17, 18] (Mendez and Cabo, 1980; Sharma et al., 2000). Recently Idika et al. [19] (2012) recorded a slightly longer prepatent period in WAD sheep but, apart from being a different species, the sheep were older than the goats used in the current study. The pathology caused was typical to that described in infections due to* H. contortus* in small ruminants and laid credence to the known fact that the parasite is very pathogenic. However, the pathology might have been severe in this study in spite of the low dose level used because of the young ages of the goats. The decision to use the low dose level (4500 L3) compared to the higher dose level used in other studies was informed by the fact that the donor WAD goats were in the same age range as those used in the current study and were passing faeces with high FEC and showed fairly low PCV. Although the animals infected with the WADHc isolate had higher FEC and WB, they coped like their counterparts infected with the RSHc, an indication that they may be more adapted to their native isolate, WADHc. The generally low FEC and WB noticed here may be partly due to the low dose level of the infective larvae used and partly due to the fact that most of the goat kids used in the study may belong to the strong responder phenotype, as 85% of the Nigerian WAD goats of the humid tropics have been shown to be naturally resistant to* H. contortus* [20, 21].

## 5. Conclusion

Both isolates of* H. contortus* were pathogenic to the WAD goats but the WAD goats seemed to be more adapted to the local isolate of* H. contortus* (WADHc) than the isolate of* H. contortus* from RS goats of northern Nigeria. The two breeds of goats could therefore be reared together since both isolates of* H. contortus* were found to be pathogenic to the WAD goats. Since both isolates were both infective and pathogenic to the WAD goats, none of them could therefore be encouraged on pastures in preference to the other as a means of controlling the infections due to* H. contortus*.

## Figures and Tables

**Table 1:** Experimental design.

Group	Number of goats	Age of goats (months)	Sex of goats	Dose level of *H. contortus* L3	Day of necropsy (after infection)
(1) Infected with RSHc isolate	10	4–6	Male	4500	56
(2) Infected with WADHc isolate	10	4–6	Male	4500	56
(3) Uninfected control	10	4–6	Male	0	56
